# Cheerios Floating on Lung Computed Tomography Scan Revealing Cholangiocarcinoma of the Lungs

**DOI:** 10.7759/cureus.2497

**Published:** 2018-04-17

**Authors:** Layth Al Attar, Aarati Keshary, Raid Abu-Awwad

**Affiliations:** 1 Internal Medicine, University of Kansas School of Medicine - Wichita

**Keywords:** lung metastases, image evaluation, cholangiocarcinoma, histology

## Abstract

Radiologic findings are important in narrowing differential diagnosis. This becomes imperative in unusual presentation of diseases. An uncommon finding on lung imaging is Cheerios signs. It is described as lesions with hypodense center and noticeable rim. These lesions are associated with bronchioloalveolar carcinoma representing lepidic growth. We present a case of rapidly worsening shortness breath and cough. Initial computed tomography scan of the chest showed cystic lesions on ground glass background in both lungs. Extensive workup for inflammatory, infectious, or connective tissue disorders was unremarkable. The biopsy of lung lesions pointed at lung metastasis of cholangiocarcinoma. Our case focuses on the benefit of imaging findings, as metastatic lesions can mimic the presentation of that of other lung diseases.

## Introduction

Computed tomography (CT) scan is fast, noninvasive, and accurate. It helps in certain clinical circumstances, can suggest a specific diagnosis, indicate a potentially treatable disease, and guide a clinician to an appropriate area for biopsy. Common features noted on CT scan are nodules, cysts, consolidations, and ground glass appearance. Physicians rely on specific imaging findings to narrow their differential diagnoses. Although bronchioalveolar carcinoma may present as bubble-like lucency of pseudocavitation referred to as Cheerios sign [1], the sign is an uncommon finding on CT scan [2-3]. We discuss a case of lung metastasis presenting with a Cheerios sign on lung imaging.

## Case presentation

**Figure 1:**
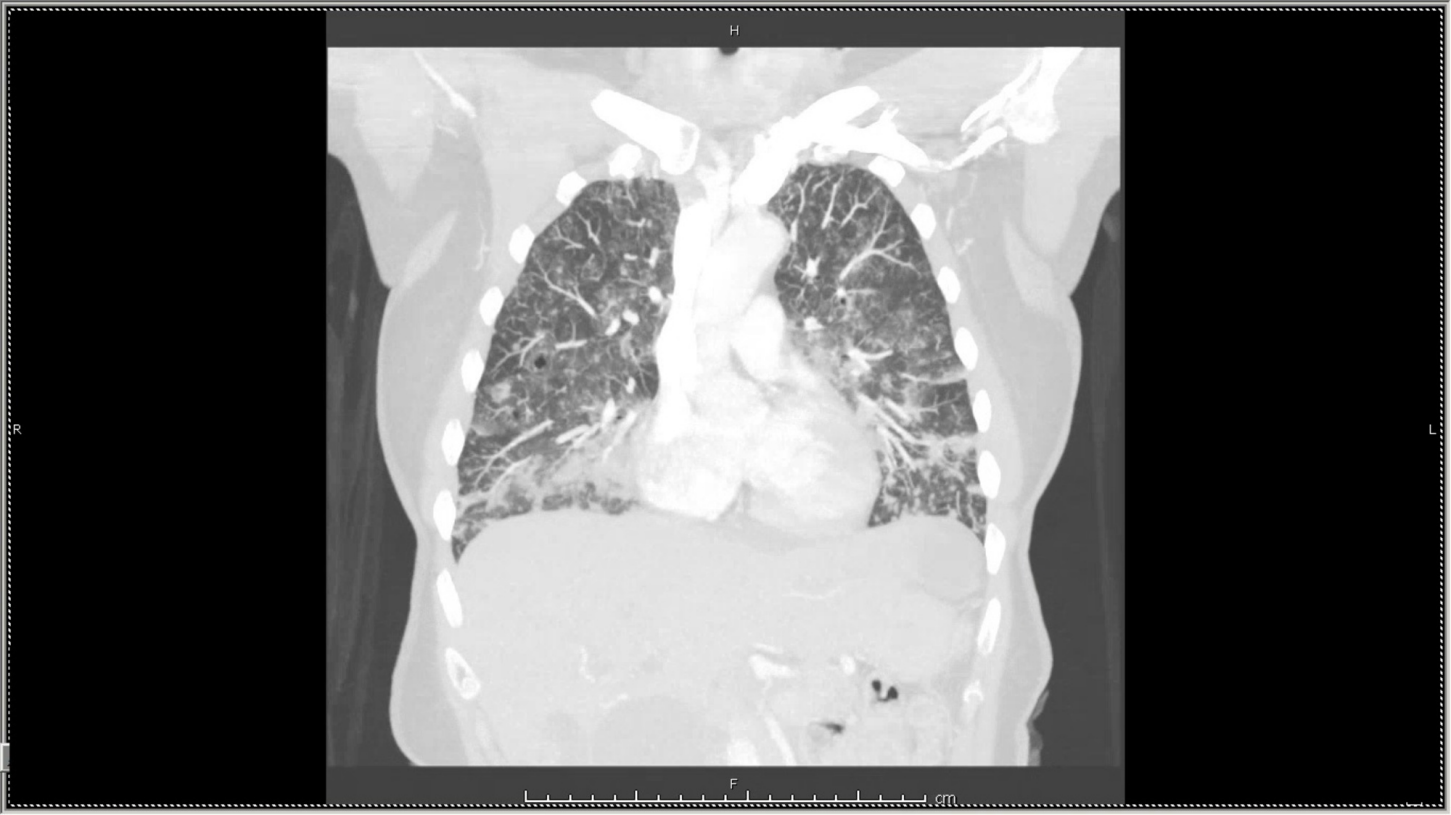
Chest Computed Tomography Scan Cheerios sign visible in lung tissue.

**Figure 2:**
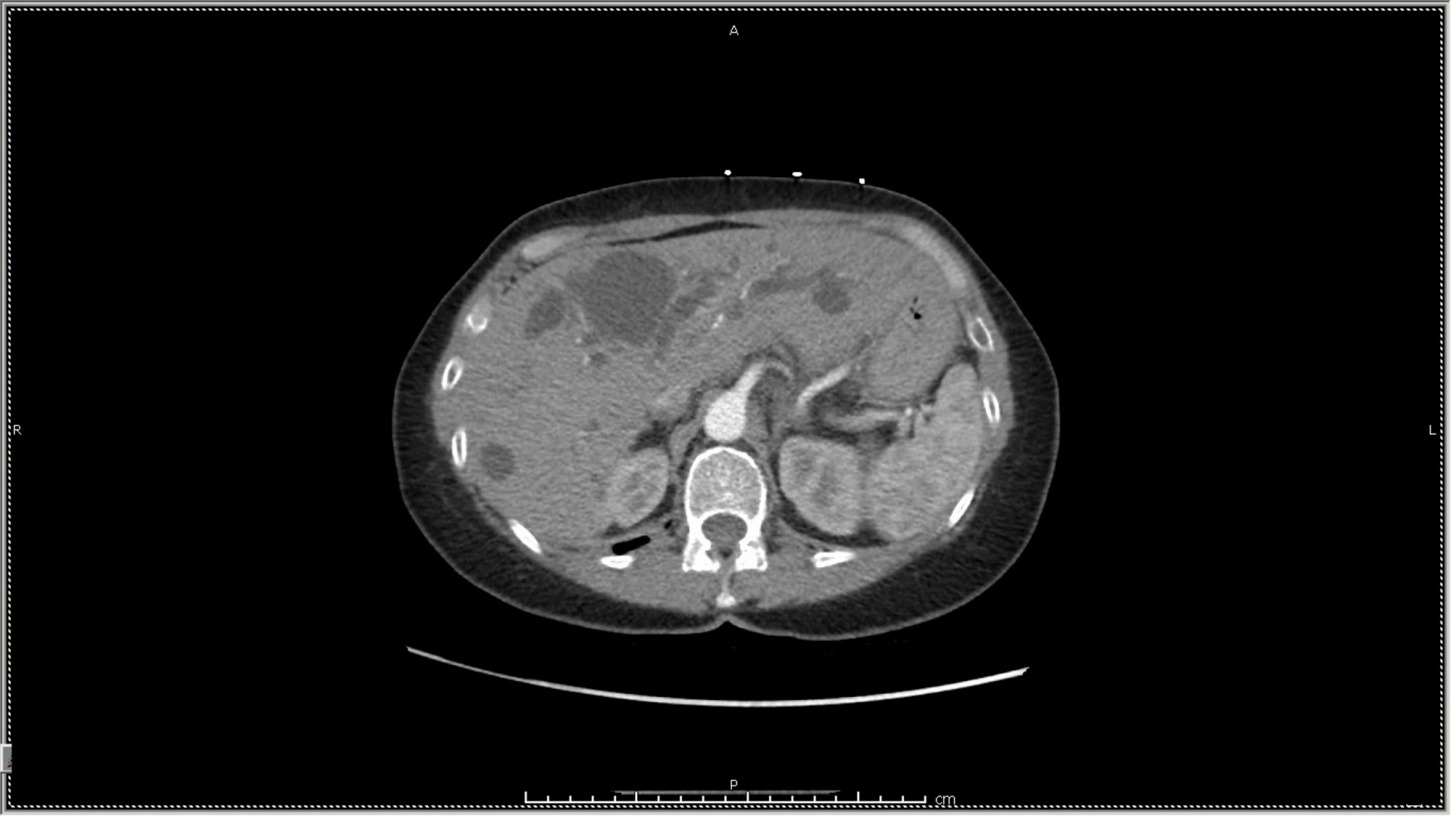
Liver Computed Tomography Scan Multiple cysts visible in the liver.

A 64-year-old female was admitted with progressively worsening dyspnea and cough of one-month duration. These symptoms were associated with an unintentional eight-pound weight loss. She was seen two weeks earlier by her primary physician and started on levofloxacin for what was thought to be pneumonia. Initial antibiotic therapy failed to improve symptoms and patient eventually required supplemental oxygen. A CT scan of the chest showed innumerable cystic lesions with diffuse ground glass opacities in both lungs and a lesion in the liver (Figure 1, Figure 2).

The patient underwent extensive workup for lung and liver disease including viral, bacterial, and fungal infection workups, human immunodeficiency virus testing, hepatitis panel, expanded connective tissue disease workup, and comprehensive interstitial lung disease markers. Cancer markers revealed an elevated cancer antigen 19-9. An abdominal ultrasound revealed innumerable cystic lesions throughout the liver; the majority were simple in appearance. It also showed intrahepatic and extrahepatic biliary and pancreatic duct dilatation. Liver biopsy was suggestive of a benign simple cyst wall and acute inflammation of hepatocytes. These findings raised a differential diagnosis including bile duct obstruction, mass effect, adjacent abscess, or adverse drug/toxin effect. Eventually, due to the unusual findings on CT scan and the failure of liver biopsy to provide a diagnosis, a lung biopsy was performed. Lung tissue was obtained by videoscopic-assisted fluoroscopic surgery to the right chest with wedge resection. Pathological examination showed metastatic with moderately differentiated adenocarcinoma in the right upper lobe, middle lobe, and lower lobe.

## Discussion

“Cheerios sign”, seen on CT-scan of the lung, is described as nodules of multiple sizes with a hypodense center and noticeable rim [2]. This appearance is due to the proliferation of cells around an airway referred to as lepidocrocite growth. They derive their name from the resemblance to the breakfast cereal, Cheerios. The differential diagnosis is wide and includes non-malignant entities such as Langerhans cell histiocytosis, lymphangioleiomyomatosis, and vasculitis as well as malignancies such as bronchioloalveolar carcinoma, metastatic adenocarcinoma, metastatic squamous cell carcinoma, and lymphoma.

Adenocarcinoma in the lung is the most common type of primary lung cancer. Lepidic growth typically is observed in bronchioloalveolar carcinoma (BAC). Lee et al. [4] described a frequent association of BAC with Cheerios sign. In addition, the lungs are a common destination for cancer spread. Common sites of original cancer are breast, gastrointestinal, ovarian, and thyroid. Usually, metastatic lesions present as pulmonary nodules and typically appear as peripheral, rounded nodules of variable size, scattered throughout both lungs [5]. Adenocarcinoma metastasis may carry a distinctive pattern, yet similar to BAC. They may grow in a lepidic fashion, with occasional air bronchograms visible. This would resemble Cheerios sign on imaging. Cheerios sign has not been reported commonly [2-3]. The true frequency of air-space pattern in lung metastasis is probably <10% [6].

A review described Cheerios sign in the setting of lung metastasis from a gastrointestinal malignancy [6]. To our knowledge, there are two reported cases of lung metastasis with cholangiocarcinoama origin. [7-8]. Air-space patterns include nodules, pulmonary consolidation, the “angiogram” sign, and Cheerios sign. This case pointed at the importance of correlating clinical findings in building a differential diagnosis. Our patient experienced a rapid decline in respiratory function, significant weight loss, and abnormal liver and lung imaging findings. Cholangiocarcinoma is not a common primary source of lung metastasis making this case unique. Recognition of Cheerios sign should raise the index suspicion for adenocarcinoma as it is a rare but characteristic finding.

## Conclusions

In conclusion, CT scan findings help limit differential diagnosis. This is achieved by recognizing specific signs associated to certain conditions. Our case was a rapidly worsening shortness of breath. The presence of Cheerios sign on CT imaging narrowed the differential to be adenocarcinoma. The specific findings on imaging studies were crucial to determine the necessity of invasive procedures for histologic diagnosis. The final diagnosis was made of uncommon lung metastasis of cholangiocarcinoma.
